# Bridging cultures: Chinese elements in scientific illustrations

**DOI:** 10.1186/s13020-024-00972-4

**Published:** 2024-07-24

**Authors:** Jianyou Gu, Wenying Zhang, Xianxing Wang, Qiang Zhou, Junfeng Zhang, Fuming Xie, Renpei Xia, Zhe-Sheng Chen, Huaizhi Wang

**Affiliations:** 1 Institute of Hepatopancreatobiliary Surgery, Chongqing General Hospital, Chongqing University, No.118, Xingguang Avenue, Liangjiang New Area, Chongqing, 401147 China; 2 Department of Nephrology, The Second Affiliated Hospital of Guangzhou Medical University, Guangzhou, China; 3 University of Chinese Academy of Sciences (UCAS) Chongqing School, Chongqing Medical University, Chongqing, China; 4 Chongqing Key Laboratory of Intelligent Medicine Engineering for Hepatopancreatobiliary Diseases, Chongqing, China; 5 Department of Pharmaceutical Sciences, College of Pharmacy and Health Sciences, St. John’s University, Queens, NY 11439 USA

**Keywords:** Scientific illustrations, Traditional Chinese Elements, Cross-cultural communication

## Abstract

In the context of globalization, the integration of cultural elements into scientific research, particularly through the incorporation of traditional Chinese cultural motifs in scientific illustrations, represents a novel frontier in enhancing the universality and appeal of scientific discoveries. This paper explores the innovative practice of embedding traditional Chinese cultural elements into scientific paper illustrations, highlighting its significant role in augmenting the global appeal of research findings, promoting diversity and innovation in scientific inquiry, and facilitating cross-cultural understanding. Through a series of case studies, including symbolic representations of ancient myths and the use of traditional themes to elucidate complex scientific phenomena, we demonstrate how this cultural integration not only makes scientific content more accessible and engaging but also fosters a deeper appreciation of Chinese heritage among international audiences. This approach not only bridges the gap between science and culture but also contributes to a more inclusive and interconnected global scientific community, underscoring the importance of cultural diversity in enriching scientific exploration and communication.

**Supplementary Information:**

The online version contains supplementary material available at 10.1186/s13020-024-00972-4.

## Background

Amidst globalization, the infusion of cultural elements into scientific discourse, notably through incorporating traditional Chinese motifs in research paper illustrations, not only diversifies scientific communication and elevates the global allure of research outcomes but also exemplifies the symbiosis of science and culture. This integration exemplifies the symbiosis of science and culture, enriching the global scientific community with unique cultural insights and fostering deeper intercultural dialogue [1].

Chinese traditional culture, with its extensive history and unique symbols, has always been a significant component of global cultural heritage. Elements such as the Yin-Yang philosophy of Tai Chi, traditional festivals and myths, the profound aesthetics of ink paintings and ancient architecture, and the visually impactful symbols of dragons and phoenixes, serve as an inexhaustible source of inspiration for researchers. These elements not only encapsulate ancient Chinese wisdom but also act as bridges linking the past and future in contemporary society. As Chinese scientific research rapidly progresses, these cultural elements are increasingly incorporated into scientific illustrations, making research findings more vivid and comprehensible, thereby enhancing the interaction and integration between science and culture.

In a globalized landscape, the barrier-free dissemination of scientific insights necessitates a balance between scientific universality and cultural diversity. The integration of traditional Chinese cultural elements thus serves not only as a cultural showcase but also as a valuable contribution to the global scientific discourse, encouraging empathy and respect across diverse research communities and establishing a platform for inclusive exchange.

Moreover, this melding signifies the increasingly indistinct boundaries between science and culture, where their convergence emerges as a pivotal driver of both scientific innovation and cultural preservation, facilitating the formation of a multifaceted global scientific community and enhancing international cultural exchanges and understanding.

Overall, the adoption of traditional Chinese cultural elements in scientific illustrations signifies an innovative stride within the realms of globalization and cross-cultural exchanges. It not only broadens the narrative scope of scientific inquiry but also promotes cultural interactions and mutual comprehension across the global scientific community, highlighting the indispensable role of cultural diversity in enriching scientific exploration. As globalization advances, the anticipated integration of more cultural elements into scientific research promises to invigorate both scientific progress and the perpetuation of cultural legacies.

## Case study

In modern scientific research, integrating symbols and stories from traditional culture can help elucidate complex scientific concepts and mechanisms. Through this approach, scientists not only gain novel perspectives but also make scientific content more accessible and communicative.

**Fig. 1:**
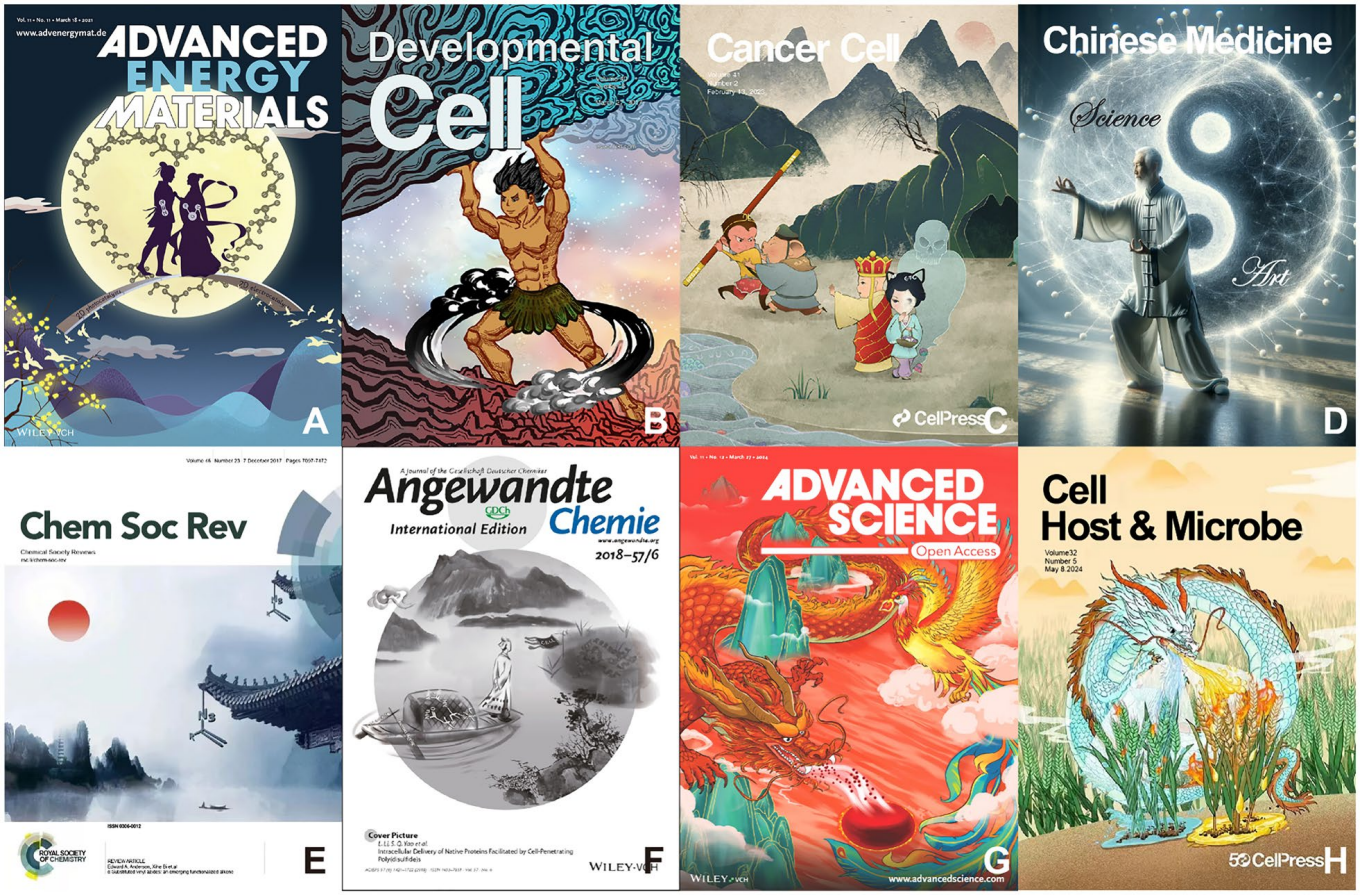
Illustrations showcasing the integration of traditional Chinese cultural elements into scientific research visuals. (Images obtained under license from the Copyright Clearance Center. The image D is produced by GPT-DALL·E.)

For instance, the ancient Chinese folk tale of the Cowherd and the Weaver Girl is ingeniously represented using two-dimensional catalysts, with N_2_ and H_2_O molecules symbolizing the celestial lovers and the magpie bridge that unites them. Under the catalytic action of these two-dimensional photocatalysts/electrocatalysts, N_2_ and H_2_O molecules are reduced to NH_3_, encircling them in a heart shape emblematic of love (Fig. 1A)[2].

Similarly, the creation myth of “Pangu-The Creator of the World” is utilized to depict how the Supramolecular Entity Complex interacts with the Notch signaling pathway in neural stem cells, thereby determining the divergent destinies of neural tissue cells and stem cells (Fig. 1B) [3].

Moreover, scientists have drawn parallels from the “Journey to the West” narrative of “Monkey Hit Lady White Bone Thrice” to elucidate how circulating tumor cells (CTCs) evade the immune system’s surveillance and attack. In this analogy, CTCs are likened to the White Bone Demon, natural killer (NK) cells to the Monkey King, and the HLA-E immune checkpoint to Pigsy, who impedes the NK cells' assault. CTCs, by consuming platelets and acquiring the RGS18 gene, successfully masquerade and elude immune detection, akin to the White Bone Demon deceiving Tang Sanzang and Pigsy, thwarting the Monkey King’s efforts (Fig. 1C) [4].

Additionally, we have endeavored to illustrate the seamless integration and intersection of science and art through the Daoist concept of the Taiji, symbolizing the harmony of Yin and Yang (Fig. 1D).

The author uses ink painting techniques to adorn the eaves with the chemical structure, seamlessly integrating it into traditional oriental architecture and natural landscapes. This symbolizes the harmonious union of science and art. The rising sun in the painting represents the vast potential of α-substituted vinyl azides as multifunctional precursors, highlighting the broad prospects and limitless possibilities in scientific research (Fig. 1E) [5].

Furthermore, the researchers used traditional Chinese ink painting to illustrate two complementary methods for endocytosis-independent intracellular protein delivery. The figure at the bow symbolizes native proteins, while the chemical structures on the boat represent the modifications and conjugates used in the delivery process. The two oars signify the main approaches of cell-penetrating poly(disulfide)s: PTM-based tagging and traceless tagging (Fig. 1F) [6].

Meanwhile, the authors metaphorically represent ferroportin, the iron-exporting pump protein, as a “Dragon,” and Hif2α activators, which enhance erroportin function, as a “Phoenix.” The phrase “Dragon and Phoenix Bring Prosperity” encapsulates their synergistic role in maintaining red blood cell health, symbolizing the treatment of refractory anemia (Fig. 1G) [7].

Additionally, the author uses a dragon to symbolize the dual functionality of the TaHRC gene. The two different alleles of TaHRC lead to distinct biological processes, resulting in varying outcomes of wheat’s susceptibility to pathogen damage (Fig. 1H) [8]. (The supplementary materials showcase the Chinese mythological stories and traditional elements, including their Chinese characters, Pinyin, corresponding English translations, and detailed narratives.)

These designs not only render the scientific content more comprehensible and engaging but also enable readers to experience the fascinating amalgamation of ancient Chinese mythology with modern technology, thereby enriching the cultural depth and visual impact of the research paper.

## The integration of scientific research and cultural values

The integration of traditional Chinese cultural elements into scientific research illustrations not only enriches the cultural depth and international appeal of research papers but also significantly fosters diversity and innovation in scientific inquiry. This profound fusion of culture and science enhances the readability and allure of the papers, offering global researchers a deeper understanding of Chinese culture and increasing international recognition of Chinese scientific endeavors.

- **Stimulating Scientific Innovation:** Cultural symbols and stories offer new metaphors and models. Drawing inspiration from traditional Chinese culture, researchers can explore novel perspectives and methodologies, breaking traditional thinking patterns and encouraging innovation. This approach not only fosters communication and mutual respect among researchers from diverse cultural backgrounds but also broadens the scope of scientific research, stimulating diverse thinking and infusing new inspiration into scientific exploration.
- **Promoting Cross-Cultural Exchange:** This trend exemplifies the integration and preservation of traditional Chinese culture, serving as a catalyst for scientific innovation by fostering cross-cultural dialogue and exploring diverse perspectives. The fusion of culture and science paves new pathways for global scientific research and cultural exchange, emphasizing the vital role of cultural diversity in advancing scientific progress and enhancing mutual understanding among different societies.
- **Diversifying Scientific Culture:** As globalization progresses, the incorporation of more cultural elements into scientific research is anticipated, bringing new perspectives and momentum to both scientific advancement and cultural heritage. The diversification of scientific culture is not only essential for scientific progress but also forms a vital foundation for cultural exchange and mutual understanding. By embracing and respecting diverse cultural elements, the scientific community can create a more open and diverse research environment, laying a solid foundation for the collective advancement of global science and technology.

In summary, integrating traditional Chinese cultural elements into scientific illustrations not only exemplifies the preservation and appreciation of Chinese culture but also opens new avenues for global scientific research and cultural exchange. This approach underscores the crucial role of cultural diversity in fostering scientific progress and promoting mutual understanding on a global scale.

## Challenges and opportunities

The increasing incorporation of traditional Chinese cultural elements in scientific research illustrations is poised to profoundly impact future scientific work, communication, and culture. However, this trend also presents a series of challenges and opportunities, necessitating a collaborative effort within the scientific community to explore and innovate continuously for the mutual advancement of scientific research and cultural heritage.

- **Challenges:** The process of promoting and deepening the use of traditional Chinese cultural elements in scientific illustrations may encounter obstacles such as cultural misunderstandings, limitations in creative expression, and inconsistencies in international acceptance. These challenges demand that researchers not only deeply understand and respect traditional culture but also possess the ability for cross-cultural communication and innovative perspectives to ensure the accurate interpretation and effective conveyance of cultural elements.
- **Opportunities:** Concurrently, this trend offers unprecedented opportunities for the scientific community. Primarily, it serves as a vital channel for the international dissemination of Chinese traditional culture. Furthermore, this cross-cultural scientific practice facilitates global scientific collaboration and cultural exchange, providing new approaches and cooperative models for addressing global scientific challenges. Lastly, it acts as a significant catalyst for scientific innovation and the development of science and technology, contributing to the cultivation of researchers with a global outlook and innovative capabilities.

## Conclusion

The integration of traditional Chinese cultural elements into scientific research illustrations not only demonstrates the harmonious coexistence of science and culture but also signifies an innovative amalgamation of scientific communication and cultural dissemination amidst the wave of globalization. This trend not only highlights the Chinese scientific community’s profound exploration and confident articulation of native culture but also reflects the global scientific community's deep respect and enthusiastic embrace of cultural diversity. By intertwining scientific inquiry with the humanities and arts, this approach imbues research findings with greater vitality and emotional resonance, significantly enriching the dimensions of scientific exchange. It fosters a deeper understanding and extensive exchange between different cultures, offering new ideas and momentum towards building a more open and inclusive global community of science and culture.

### Supplementary Information

Additional file 1.

## Data Availability

Not applicable.

## Declarations

### Declaration of AI and AI-assisted technologies in manuscript preparation processes

During the preparation of this manuscript, the authors utilized ChatGPT for language refinement and GPT-DALL·E for the creation of illustrative diagrams. Following the application of these artificial intelligence tools, the authors conducted a thorough review and necessary adjustments to the text and graphical content, thereby assuming full responsibility for the publication's content.
