# Correction: Bridging cultures: Chinese elements in scientific illustrations

**DOI:** 10.1186/s13020-024-00985-z

**Published:** 2024-08-29

**Authors:** Jianyou Gu, Wenying Zhang, Xianxing Wang, Qiang Zhou, Junfeng Zhang, Fuming Xie, Renpei Xia, Zhe-Sheng Chen, Huaizhi Wang

**Affiliations:** 1 Institute of Hepatopancreatobiliary Surgery, Chongqing General Hospital, Chongqing University, No.118, Xingguang Avenue, Liangjiang New Area, Chongqing, 401147 China; 2 Department of Nephrology, The Second Affiliated Hospital of Guangzhou Medical University, Guangzhou, China; 3 University of Chinese Academy of Sciences (UCAS) Chongqing School, Chongqing Medical University, Chongqing, China; 4 Chongqing Key Laboratory of Intelligent Medicine Engineering for Hepatopancreatobiliary Diseases, Chongqing, China; 5 Department of Pharmaceutical Sciences, College of Pharmacy and Health Sciences, St. John’s University, Queens, NY 11439 USA

**Correction: Chinese Medicine (2024) 19:103** 10.1186/s13020-024-00972-4

Following publication of the original article [1], the authors reported that the corresponding author Zhe-Sheng Chen was not marked, and the corresponding author information was missing. The correct author information has been provided in this Correction.

The original article [1] has been corrected.
