# Supplementary material for: Bridging cultures: Chinese elements in scientific illustrations
Source: Chin Med. 2024 Jul 24;19:103. doi: 10.1186/s13020-024-00972-4 (PMC11267676; doi:10.1186/s13020-024-00972-4)
Supplement: Supplementary file 1 — Additional file 1. [file 13020_2024_972_MOESM1_ESM.docx]

**Chinese Mythological Stories and Traditional Elements in Chinese, Pinyin, and English Translation with Stories**

**牛郎织女**

Chinese: 牛郎织女

Pinyin: Niúláng Zhīnǚ

English Translation: The Cowherd and the Weaver Girl

Story: Niulang (Cowherd) was a cowherd youth, and Zhinu (Weaver Girl) was a celestial maiden who weaved beautiful clouds in the heavens. They met and fell in love, but the Jade Emperor forbade their union, separating them on opposite sides of the Milky Way. Once a year, on the seventh day of the seventh lunar month, magpies form a bridge over the Milky Way, allowing them to reunite briefly.

**盘古开天辟地**

Chinese: 盘古开天辟地

Pinyin: Pángǔ Kāi Tiān Pì Dì

English Translation: Pangu - The Creator of the World

Story: Pangu is the creator god in ancient Chinese mythology. He used a giant axe to split the chaos, allowing the light, clear air to rise and form the heavens, and the heavy, turbid air to sink and form the earth. Pangu stood between them, growing taller as they separated. After the heavens and earth were fully formed, Pangu exhausted himself and died. His body transformed into various elements of nature.

**孙悟空三打白骨精**

Chinese: 孙悟空三打白骨精

Pinyin: Sūn Wùkōng Sān Dǎ Báigǔ Jīng

English Translation: Monkey Hit Lady White Bone Thrice

Story: In "Journey to the West," Tang Sanzang and his disciples encounter the demon White Bone Spirit while traveling to retrieve Buddhist scriptures. The demon changes her appearance multiple times to deceive Tang Sanzang, but each time, Sun Wukong (Monkey King) sees through her disguises and defeats her. This story symbolizes the steadfast belief in justice triumphing over evil.

**道教**

Chinese: 道教

Pinyin: Dàojiào

English Translation: Daoism

Background: Daoism is an indigenous Chinese religion that originated from the reverence of nature and the cosmos. Daoism emphasizes harmony between humans and nature and advocates living in accordance with natural principles.

**太极**

Chinese: 太极

Pinyin: Tàijí

English Translation: Tai Chi

Background: Tai Chi is a significant concept in Daoism, symbolizing the origin of the universe and the foundation of all things. The Tai Chi symbol, a circle with black and white interlocking shapes, represents the harmony and unity of Yin and Yang. Yin and Yang are two opposing yet complementary forces in the universe, and the Tai Chi symbol illustrates the dynamic balance and the process of creation from nothingness.

**阴阳**

Chinese: 阴阳

Pinyin: Yīnyáng

English Translation: Yin and Yang

Background: Yin and Yang are fundamental concepts in ancient Chinese philosophy, representing two opposing yet complementary forces in nature and human society. Yin signifies darkness, softness, femininity, and calmness, while Yang signifies light, strength, masculinity, and intensity. The interaction and interdependence of Yin and Yang drive the changes and development in the world.

**水墨画**

Chinese: 水墨画

Pinyin: Shuǐmòhuà

English Translation: Ink Painting

Background: Ink painting is a traditional Chinese art form that uses water and ink as its primary materials. Artists employ various brush techniques and varying ink tones to depict objects and convey emotions. Ink painting emphasizes creating an artistic conception and spirit, aiming for an artistic effect that lies between likeness and unlikeness, often featuring landscapes, flowers, birds, and human figures.

**龙**

Chinese: 龙

Pinyin: Lóng

English Translation: Dragon

Background: The dragon is a mythical creature in Chinese mythology, symbolizing power, authority, and good fortune. It is often depicted as a long, scaled, four-clawed, and water-spouting animal capable of flying. In Chinese culture, dragons are believed to control rain and water, are considered the emperor's emblem, and are seen as auspicious symbols.

**凤凰**

Chinese: 凤凰

Pinyin: Fènghuáng

English Translation: Phoenix

Background: The phoenix is a divine bird in Chinese mythology, symbolizing nobility, auspiciousness, and immortality. It is depicted with beautiful feathers and an elegant posture, frequently appearing in legends and art. The phoenix often pairs with the dragon, representing the harmony of Yin and Yang and the balance of heaven and earth.

**龙凤呈祥**

Chinese: 龙凤呈祥

Pinyin: Lóng Fèng Chéng Xiáng

English Translation: Dragon and Phoenix Bring Prosperity

Background: "Dragon and Phoenix Bring Prosperity" is an auspicious motif in Chinese culture, symbolizing the harmony of Yin and Yang, marital bliss, and family prosperity. The dragon represents Yang, while the phoenix represents Yin. Together, they signify a harmonious and prosperous life, often used in weddings and celebrations to bless the couple and express hopes for a happy life.
